# Influence of cannabis potency on mental health outcomes among adolescents and young adults: scoping review

**DOI:** 10.1192/bjo.2026.12023

**Published:** 2026-07-07

**Authors:** Chloe Hambly Lapointe, Lilith Nosko, Candice E. Crocker, Philip G. Tibbo

**Affiliations:** Research, Innovation and Discovery, Nova Scotia Health Authority, Halifax, Canada; Psychiatry, Dalhousie University, Halifax, Canada; Diagnostic Radiology, Dalhousie University, Halifax, Canada

**Keywords:** Cannabis, potency, young adult, adolescent, mental health disorders

## Abstract

**Background:**

Recently, there has been a significant rise in potency (% tetrahydrocannabinol (THC)) of cannabis products globally. As such, there is a need for a better understanding of the relationship between cannabis potency and mental health outcomes, especially in a developmentally vulnerable population such as adolescents and young adults.

**Aims:**

The objective of this scoping review was to summarise existing literature investigating the potency of cannabis products as it relates to mental health outcomes in adolescents and young adults aged 14–25.

**Method:**

Systematic searches of MEDLINE, Embase, CINAHL and PsycINFO were conducted for relevant manuscripts up to October 2025. Following PRISMA-ScR guidelines, retrieved studies were then screened and data extracted by two independent reviewers.

**Results:**

Out of 11 225 studies identified by our searches, 71 were included in the review after screening. Compared with low-potency cannabis, our findings suggest that high-potency cannabis is more strongly associated with severe mental health issues, such as cannabis dependence, psychosis and cognitive deficits.

**Conclusions:**

Overall, it was found that high-potency cannabis use (>15% THC) was associated with a great number and magnitude of adverse mental health outcomes. As such, the potency of cannabis products should be measured in future cannabis research that investigates short- and long-term outcomes. Additionally, the potency of cannabis products should be a consideration in any future cannabis regulatory policy discussions.

Cannabis is one of the most widely consumed psychoactive substances globally, with an estimated 219 million worldwide users as of 2021.^1^ Globally, there has been an increase in the concentration of the primary psychoactive compound in cannabis: tetrahydrocannabinol (THC), especially in North America and Europe.^2^

In the USA, the average THC concentration of federally seized cannabis increased from 8.9% in 2008 to 17.1% in 2017.^3^ Similar trends are reported by potency monitoring programmes in the European Union, which found that the average THC concentration of herbal cannabis increased from 5.0% in 2006 to 10.2% in 2016.^4^ In Canada, the THC content of dried cannabis legally available has risen sharply in the past two decades, increasing from an average of 4% THC in the 2000s to 20% in 2023.^5^ The most frequently purchased cannabis products available at retailers across Canada often have a potency starting as high as 20–30% THC.^6,7^

Cannabis use has both acute and long-term impacts on mental health. Acute cannabis use is associated with transient cognitive impairment,^8^ anxiety^9–12^ and psychotic-like experiences,^13–15^ while chronic cannabis use is associated with other substance dependence.^16^ Chronic cannabis use alone, as well as other co-occurring substance use, is associated with the development, or worsening, of mood and psychotic disorders.^17^ Further research suggests that these aforementioned effects could be moderated by cannabis potency, such that an increased THC content is associated with a greater magnitude of use consequences, such as cognitive deficits^18,19^ and anxiety.^12,20^ Despite its importance, the influence of cannabis potency on mental health outcomes is often not explicitly examined, making its impact unclear.

Determining how cannabis potency affects users is especially important when considering adolescent and young adult (AYA) populations. Compared with older users, AYAs are at a higher risk of experiencing long-term consequences of substance use due to the critical neurodevelopmental time period coincident with this age range.^21^ Cannabis use can cause disruptions to the brain’s endocannabinoid system, which has an important role in brain maturational processes during adolescence,^22,23^ potentially resulting in a variety of mental health issues.^23^ AYAs use cannabis more frequently than other age groups,^24^ and use cannabis of a higher THC potency than previous generations. As such, a better understanding of high cannabis potency effects in AYAs is needed.

A recent scoping review by Bero et al assessed the health effects of high-potency cannabis (HPC) products across various age groups.^25^ The authors reported that studies assessing the mental health consequences of HPC were the most common in the literature; however, the specific consequences were not explicitly reported on. In addition, even though AYA made up a portion of their sample, Bero et al’s lack of focus on – or restriction to – an AYA age range makes their results less accessible to parties interested in determining how cannabis potency affects AYA mental health. Another existing review on the health impact of cannabis potency limited their focus to mental health disorders.^26^ However, this review only discussed select mental disorders, and like Bero et al, Petrilli et al’s review did not specifically assess an AYA age range.^25,26^ Consequently, while being effective explorations of their respective topics, these existing reviews are not best equipped to address the question of the effect of cannabis potency on cannabis-related mental health outcomes in AYA populations. Addressing this question is vital to understanding the impact of cannabis potency on a developmentally vulnerable age group. Understanding this relationship will also inform future cannabis-potency research and foster the subsequent development of public health strategies and targeted interventions. This gap in knowledge necessitates a precise yet thorough summary of the existing evidence.

To address this need, we conducted a scoping review to provide a broad overview of the literature, rather than attempting to answer a specific question.^27^ As such, our scoping review aimed to identify and summarise existing literature regarding the relationship between cannabis potency and the onset or progression of mental health conditions, with a focus on individuals aged 14–25. The present review also sought to explore potential differences in this relationship based on the gender and/or sex of the user.

## Method

This review was conducted in accordance with the JBI methodology for scoping reviews and Lockwood et al’s scoping review methodological framework.^27,28^ The protocol for this review was registered *a priori* in Open Science Framework^29,30^ (https://doi.org/10.17605/OSF.IO/9R7QC).

### Eligibility criteria

This review included peer-reviewed studies, preprints and theses/dissertations that reported the effect that cannabis of a specific potency had on the mental health outcomes of AYAs. We defined AYAs as individuals between the ages of 14 and 25. This age range was chosen due to its accordance with the World Health Organization’s definition of AYA^10–24,31^, and the age by which puberty usually begins.^32^ Studies involving cohorts outside this age range were included only if the source could establish that the participants had used cannabis within the specified age range.

Reporting of cannabis potency included by percentage, mass and ratio of cannabinoids, and reported via self-report or laboratory-based analyses. Studies reporting exclusively on synthetic and medical-grade cannabis (i.e. dronabinol) were excluded. When author-given potency stratification was not used, we categorised cannabis as high potency if it contained 15% or more THC (15 mg/g) and as low potency if it contained less than this amount. Studies reporting on recreational and publicly available cannabis were included for this review. Studies exclusively reporting on epilepsy, somatic, motor or physical sensation related outcomes were excluded from the present review.

### Search strategy

We conducted our searches of Ovid MEDLINE (NLM, Wolters Kluwer), CINAHL (EBSCO), PsycINFO (American Psychological Association) and Embase (Elsevier) on 3 June 2024, with a secondary search on 6 October 2025, to include eligible sources published since the primary search. The terms used in these searches are included in Appendix A. Sources derived from these searches were then collated and uploaded into Covidence Systematic Review Software (Veritas Health Innovation, Melbourne, Australia; www.covidence.org) for Windows. Duplicate sources were removed both automatically by the review software and manually by a reviewer (C.H.L.).

### Study selection

**Fig. 1:**
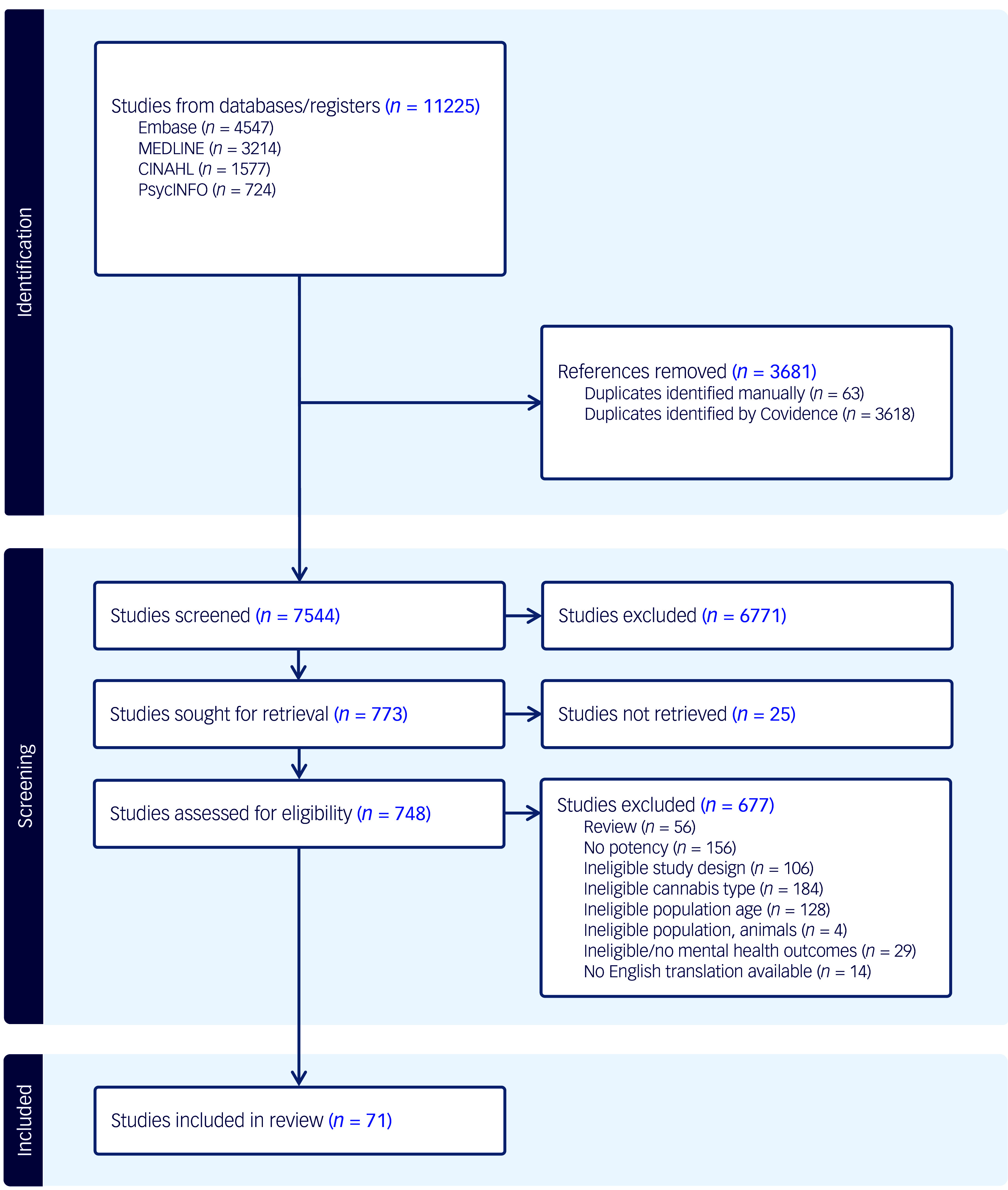
Fig. 1 long description. PRISMA-ScR diagram.

Once in Covidence, sources derived from the searches were screened twice. First, two independent reviewers screened the titles and abstracts of all sources (C.H.L. and L.N.). Sources which were found to meet the inclusion criteria at this point then had their entire text reviewed by the same two reviewers for further confirmation of the source’s suitability for the current review. Reference lists of reviews that made it to the full-text stage were manually screened for relevant sources. Any time a source was excluded from the review, the reason for doing so was recorded and are presented in the Preferred Reporting Items for Systematic reviews and Meta-Analyses extension for Scoping Reviews (PRISMA-ScR) flow diagram (Fig. 1). If at any point there was confusion or a conflict between the reviewers that could not easily be resolved, a third reviewer would assess the source (C.E.C. or P.G.T.).

### Data extraction

Data were extracted by two independent reviewers (C.H.L. and L.N.) using an extraction tool developed by C.H.L. in Covidence. A pilot test was conducted with five studies to ensure the data-extraction sheet included all necessary variables and outcomes. Ambiguities were resolved again through consultation with a third reviewer (C.E.C. or P.G.T.). The extraction sheet used included study design characteristics, aims, participant information, cannabis-use patterns and mental health outcomes.

### Sex and gender considerations

Our secondary objective was to assess if gender and/or sex moderated the relationship between cannabis potency and AYA mental health outcomes. For the purposes of this review, we defined sex as being an individual’s biological characteristics present from birth, and gender as the roles and identities that an individual assumes regardless of biological characteristics.^33^ Included studies which assessed gender and/or sex in relation to cannabis and AYA mental health outcomes had these additional findings extracted.

## Results

### Search results

In total, 11 225 studies were identified across our initial and updated searches and imported into Covidence, 7544 of which remained after duplicates were removed. Of these studies, 773 moved forward to full-text screening where a further 677 were excluded and 25 were unable to be retrieved due to having no available full-text manuscript. After this screening process and citation searching, 71 studies remained which had relevant data extracted. The screening process is illustrated in the PRISMA-ScR flow diagram (Fig. 1).

**Fig. 2:**
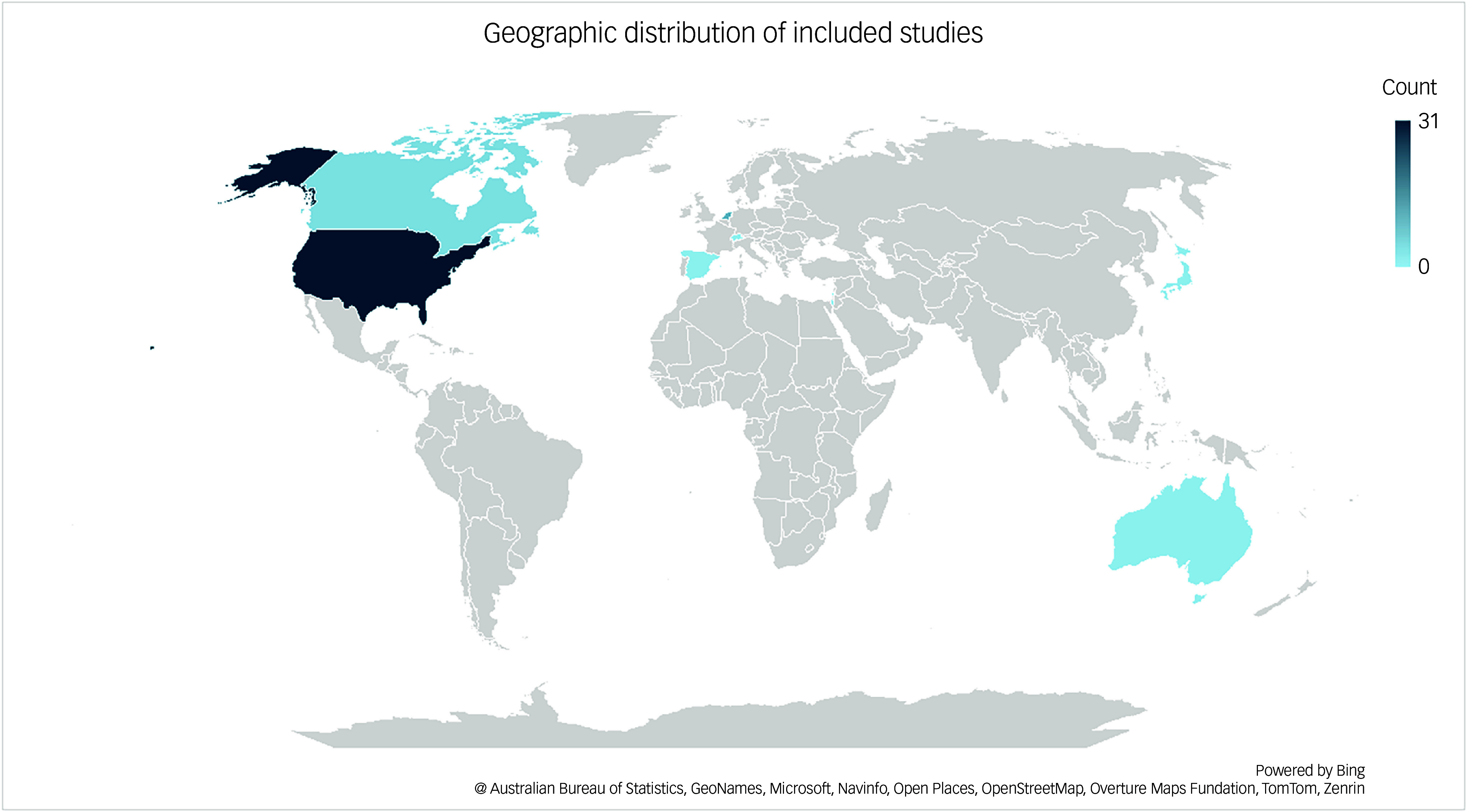
Location of where the included studies were conducted, excluding studies conducted across multiple nations.

The studies included in this review encompass a broad time frame, with publication dates spanning from 1979 to 2025. However, analysis of the included studies reveals a geographical concentration, with approximately 44% of the included research being conducted in the USA, 21% in England and 11% in the Netherlands (see Fig. 2). Regarding regulatory contexts at the time of publication, 27% of the studies were carried out in nations where cannabis was only legal for medical use. In contrast, 18% of the research was conducted in areas where cannabis was legal for recreational use, and 53% in locations with a mixed regulatory status (i.e. legal in some jurisdictions within a country but not others).

The results presented in this review are highly heterogeneous with respect to reported cannabis potency across studies. Studies were organised according to their classification by cannabis potency, focus on either long-term or acute effects and study design (Table 1). Of the total studies reviewed, 34 (47.9%) studies focused on the long-term effects of HPC with 7 (20.6%) utilising longitudinal designs (Table 2), 13 (38.2%) utilising a cross-sectional design with quantitative measures of potency (Table 3), and 14 (41.2%) utilising a cross-sectional design with qualitative measures of potency (Table 4). Eight (11.3%) included studies reported on the long-term effects of low-potency cannabis (LPC) (Table 5). The remaining, comprising 29 studies (40.8%; 6 HPC, 23 LPC), reported on the acute effects of cannabis (Table 6). The remainder of this review summarises this literature. Results regarding the acute effects of LPC use^34–56^ are present in the Supplementary Table S1 available at https://doi.org/10.1192/bjo.2026.12023.

**Table 1:** Distribution of included studies by potency, time frame and design categories

Included study classification by potency, time frame and design	*n* (%)
Long-term effects of HPC	34 (47.9)
Longitudinal	7 (20.6)
Cross-sectional (quantitative)	13 (38.2)
Cross-sectional (qualitative)	14 (41.2)
Long-term effects of LPC	8 (11.3)
Acute effects	29 (40.8)
High potency	6 (20.7)
Low potency	23 (79.3)

HPC, high-potency cannabis; LPC, low-potency cannabis.

**Table 2:** Findings from included studies with results regarding the long-term effects of high-potency cannabis (longitudinal)

Ref. #	Study design	Sample	Age (mean (s.d.), range)	Cannabis potency (% THC)	Findings
(57)	Longitudinal (baseline and 6-month assessment)	73 occasional cannabis users and 82 regular cannabis users	21.1 (2.32)	Flower: 15–17% Hash-oil: 80–90%	Increased frequency of hash-oil was associated with more problematic cannabis use and negative cannabis-related outcomes at follow-up.
(58)	Longitudinal	338 using sexual minority and gender diverse women	22.25 (2.04; 18–25)	Categorical self-report	Use of cannabis concentrates was associated with an increased number of cannabis use disorder criteria met.
(59)	Longitudinal	863 adult cannabis users	NR (NR; 20–24)	195 pg/mg (s.d. = 403) of THC in hair samples	Greater THC exposure at age 20 was associated with increased aggression, psychotic-like experiences, internalising symptoms and problematic substance use at age 24 as well as reduced quality of life and occupational functioning.
(60)	Longitudinal	338 cannabis using sexual and gender minority women	22.25 (2.04; 18–25)	Categorical self-report	Use of cannabis concentrates was not associated with problematic cannabis use outcomes.
(61)	Prospective cohort	269 cannabis users	Transitioned to dependence: 21.9 (NR) No transition: 21.8 (NR)	Categorical self-report	Cannabis potency was not associated with the likelihood of developing cannabis dependence.
(62)	Case-control	410 patients with FEP, 370 controls	FEP: 27.1 (8.7) Controls: 30.0 (9.0)	Categorical self-report	Regular use of high-potency cannabis was associated with an increased risk of psychotic disorders compared to those who had not used cannabis.
(63)	Case-control	802 patients with FEP, 1263 controls	FEP: 30.5 (10.3) Control: 36.1(13.0)	Categorical self-report	Patients with FEP who used high-potency cannabis regularly were more likely to be in the deteriorating subgroup of patients.

THC, tetrahydrocannabinol; NR, not reported; FEP, first episode of psychosis.

**Table 3:** Findings from included studies with results regarding the long-term effects of high-potency cannabis (cross-sectional with quantitative potency)

Ref. #	Study design	Sample	Age (mean (s.d), range)	Cannabis potency %THC	Findings
(64)	Cross-sectional	121 users 152 non-users	Users: 20.6 (4.3) Non-users: 21.1 (4.8)	Hash oil: ≥50–60%	After accounting for all covariates, hash-oil use was associated with increased cannabis dependence in a dose-dependent manner (RR = 1.2).
(65)	Cross-sectional	Non-users: 31 463 Non-concentrate: 4379 Concentrate users: 11 300	Non-users: 14.94 (s.e. = 0.07) Non-concentrate: 15.17 (s.e. = 0.07) Concentrate: 15.15 (s.e. = 0.07)	Non-concentrate:12–20% Concentrate: >39%	Use of cannabis concentrates was associated with increased non-cannabis substance use compared to both non-concentrate cannabis users and cannabis non-users.
(66)	Cross-sectional	47 511 cannabis users	25.03 (8.85)	Herbal: 6–9% Sinsemilla: 17% Hashish: 15–20% Concentrates: ≥70–80%	Use of high-potency cannabis products (sinsemilla and hashish) was associated with a higher risk of cannabis dependence than the exclusive use of low-potency cannabis.
(67)	Cross-sectional	617 cannabis users	19.22 (1.42)	Herbal: 22.62% (s.d. = 7.59)	Higher-potency herbal cannabis use was associated with a higher number of CUD symptoms (IRR = 1.01).
(68)	Cross-sectional	99 cannabis users	20.32 (3.62)	Flower/bud: 19.6% Concentrate: 68%	No association between concentrate use and problematic cannabis use.
(69)	Cross-sectional	300 cannabis users	34.81 (14.90)	Flower: 15–20% Edible: 31.36 mg (s.d. = 40.76) Concentrate: 60–80%	Potency of herbal cannabis and edibles was not associated with CUD symptomology. Use of higher-potency concentrates was associated with reduced cannabis withdrawal.
(70)	Cross-sectional	70 adult cannabis users (flower: 32, concentrates: 30, other product: 8) 24 non-users	Flower: 18.7 (0.6; 18–20) Concentrates: 18.8 (0.7; 18–20) Other: 19.0 (1.3; 18–21) Control: 19.7 (0.8; 18–21)	Flower: 0.3–43.8% THC Concentrates: 40–98.5% THC THC-COOH in hair: 0.03–7.8 pg/mg	High-potency cannabis use was not associated with problematic cannabis use measures.
(71)	Cross-sectional	Herbal: 69 Skunk: 30 Mixed: 45 None: 323	Herbal: 16.9(0.85) Skunk: 17.0 (0.81) Mixed: 16.8 (0.76) None: 16.8 (0.76)	Grass: 3–4% Hashish: 6–7% Sinsemilla/skunk: 13–15% Concentrate: 76%	High-potency cannabis (skunk) users reported experiencing paranoia more often than low-potency cannabis (herbal) users (OR = 2.45, 95% CI = 1.29–4.63). Mixed-potency cannabis users reported experiencing more difficulties with sleep than non-users.
(72)	Cross-sectional	High CBD users: 1214 Low CBD users: 663	High CBD users: 23.1 (4.4) Low CBD users: 24.3 (7.0)	High CBD: 0.3–8.8% Low CBD: 0.2%	Use of higher % CBD cannabis was associated with a decreased prevalence of positive psychotic symptoms.
(73)	Cross-sectional	3389 adult cannabis users (past users: 816, current users: 2573)	Current: 29.73 (9.15) Past: 34.68 (10.79) Controls: 33.93 (11.69)	Standard THC units: 206 (s.d. = 268)	Higher THC intake was associated with increased paranoia. Among those with traumatic childhood experiences, those who consumed more THC experienced significantly more paranoia than those who consumed less THC.
(74)	Cross-sectional	280 patients with FEP and 174 controls	Patients: 25 (6.9) Controls: 27 (5.6)	Resin (hash): 3.9% Imported herbal: 1% Sinsemilla (skunk): 12–18%	FEP more likely to regularly use high-potency cannabis than controls (78 *v*. 31% of sample) with daily use of high-potency cannabis significantly associated with FEP group (OR = 12.1, 95% CI = 3.7–37.3). Non-daily use of high-potency cannabis was also associated with an increased likelihood of FEP (OR = 5.7, 95% CI = 2.5–12.6).
(75)	Cross-sectional	156 cannabis users	Flower users: 31.1 (6.5) Concentrate users: 29.8 (7.1) Mixed users: 30.4 (7.1)	Flower: 20% (s.d. = 6.3) Concentrate: 76% (s.d. = 13.2)	Increased concentrate potency was associated with an increase in the number of mental health symptoms related to depression (*r* = 0.17), anger (*r* = 0.21), mania (*r* = 0.25), anxiety (*r* = 0.21), psychosis (*r* = 0.11), sleep problems (*r* = 0.14), repetitive thoughts and behaviours (*r* = 0.10) and personality (*r* = 0.18).
(76)	Cross-sectional	123 adolescent users 123 none	Users: 14.3 (0.7; 12.8–15.8) Non-users: 14.5 (0.7)	THC-COOH in hair: mean 6.2 pg/10 mg (s.d. = 17.9; 0–103)	Greater past-year THC intake was significantly associated with worsened response inhibition and vocabulary. Hair samples: mean 42.7 pg/mg (s.d. = 88.0; 0–497).

THC, tetrahydrocannabinol; RR, rate ratio; CUD, cannabis use disorder; IRR, incidence rate ratio; THC-COOH, 11-nor-9-carboxy-THC; OR, odds ratio; CBD, cannabidiol; FEP, first episode of psychosis.

**Table 4:** Findings from included studies with results regarding the long-term effects of high-potency cannabis (cross-sectional with qualitative potency) Table 4 long description.

Ref #	Study design	Sample	Age (mean (s.d.), range)	Cannabis potency	Findings
(77)	Cross-sectional	1390 cannabis-using college students	19.84 (1.34, 18–24)	Categorical self-report	Primarily concentrate cannabis users had significantly higher scores on the CUDIT-R but endorsed similar cannabis use consequences as the class of primarily flower users.
(78)	Cross-sectional	695 adult cannabis users (474 flower, 139 concentrate, 82 edible)	Flower users: 27.22 (6.04) Concentrate: 25.97 (4.92) Edible users: 27.43 (7.55)	Categorical self-report	Use of high-potency cannabis use was significantly associated with increased problematic cannabis use. Age of cannabis-use onset: flower users: 22.45 (5.59; concentrate users: 22.30 (5.20); edible users: 22.96 (5.92)).
(79)	Cross-sectional	387 cannabis users (230 flower users, 157 concentrate users)	Flower users: 19.1 (1.6) Concentrate users: 18.4 (0.8)	Categorical self-report	Use of high-potency cannabis was associated with a greater prevalence of negative outcomes related to cannabis use (i.e. blackouts, social/interpersonal consequences, risky behavior, diminished self-perception, lack of self-care, academic/occupational consequences, impaired control and dependence symptoms).
(80)	Cross-sectional	69 adolescent cannabis users	16.4 (12–18)	Categorical self-report	High-potency cannabis use was associated with an increased likelihood of CUD diagnosis.
(81)	Cross-sectional	2514 cannabis users	24.25 (6.86)	Categorical self-report	Use of high-potency cannabis was associated with an increased number of cannabis dependence symptoms and with experiences of paranoia and deficits in memory.
(82)	Cross-sectional	71 cannabis users with psychiatric conditions	35.1 (10.2)	Categorical self-report	Use of high-potency cannabis was associated with an increased risk of developing a cannabis dependence disorder and a decreased risk of developing a substance-use-related psychotic disorder.
(83)	Cross-sectional	74 cannabis users	Median (MAD): 21 (2.0)	Ordinal and categorical self-report	Use of higher-potency cannabis was associated with an increased number of cannabis dependence symptoms endorsed and an increased prevalence of withdrawal and craving.
(84)	Cross-sectional	1007 cannabis users	19.2 (0.8)	Categorical self-report	Use of higher-potency forms of cannabis was associated with an increased risk of experiencing an increase in cannabis-dependence symptoms.
(85)	Cross-sectional	1412 students who use cannabis 4953 matched student non-users	THC only: 19.69 (2.55) CBD only: 20.31 (3.12) THC + CBD: 20.36 (3.01) Non-users: 19.20 (2.56)	Categorical self-report	Compared to using cannabis with little or no THC, use of cannabis with a higher THC content was associated with increased polysubstance use, problematic alcohol use, past-year binge drinking and past-month tobacco, alcohol and e-cigarette use. Compared to CBD and THC co-use, THC-only cannabis use was associated with a lower age of tobacco-, alcohol- and THC-use onset.
(86)	Cross-sectional	4669 students (2485 cannabis)	19.62 (2.05)	Categorical self-report	Cannabis potency was not associated with any mental health outcome.
(87)	Cross-sectional	209 using adolescents (low potency: 38, high potency: 171) 6402 non-users	Low potency: 13.75 (0.37) High potency: 13.76 (0.39) Control: 13.69 (0.40)	Categorical self-report	Compared to cannabis non-users, use of high-potency cannabis was associated with an increased likelihood of reporting a probable anxiety disorder AOR = 1.45, 95% CI: 0.96–2.20), depressive disorder (AOR = 1.59, 95% CI: 1.06–2.39) and auditory hallucinations (AOR = 1.56, 95% CI: 0.98–2.47).
(88)	Cross-sectional	410 FEP (256 users, 154 non-users)	Users: 28.2 (8.0) Non-users: 31.4 (9.9)	Categorical self-report	Cannabis use of any potency was associated with an earlier onset of psychosis, with regular use of high-potency cannabis use being associated with the earliest age of onset.
(89)	Cross-sectional	Inpatient adolescents vaping: 64 Non-vaping use: 144 None: 262	Vaping: 15.56 (1.59) Non-vaping: 15.72 (1.42) Non-users: 14.47 (1.69)	Categorical self-report	More frequent use of high-potency cannabis was associated with an increased risk of suicide attempts in the 30 days prior to admission (AOR = 2.38, *p* = 0.002).
(90)	Cross-sectional	29 cannabis users	20.86 (3.71)	Self-report	Use of high-potency cannabis was associated with worsened verbal fluency and verbal memory and cognitive symptoms similar to those with prodromal psychotic disorders.

CUDIT-R, The Cannabis Use Disorder Identification Test – Revised; CUD, cannabis use disorder; MAD, median absolute deviation; THC, tetrahydrocannabinol; CBD, cannabidiol; AOR, adjusted odds ratio; FEP, first episode of psychosis.

**Table 5:** Findings from included studies with results regarding the long-term effects of low-potency cannabis Table 5 long description.

Author (year)	Study design	Sample size	Age (mean, s.d., range)	Cannabis potency (%THC)	Findings
(91)	Longitudinal (time points: 3-year intervals ages 12–14 to ages 24–26; annual assessments ages 11–26)	527 cannabis users	NR	National average of 3.50% in 1994 to 12.30% in 2012	As the national average cannabis potency increased, the risk of new users transitioning to experience CUD symptoms increased (HR = 1.36, 95% CI = 1.20–1.54) when controlling for daily use, birth year and sex. When controlling for regular use instead of daily use, this risk of later developing CUD symptoms increased (HR = 1.41, 95% CI = 1.24–1.60).
(92)	Cross-sectional study	14 cannabis users	20.4 (1.3)	8.6% (4.0)	All participant scores on the AES-S were greater than the general population mean.
(93)	Cross-sectional	1087 cannabis users	NR	High potency: ≥10% Low potency: <10%	Use of high-potency cannabis was associated with an increased risk of experiencing generalized anxiety disorder (AOR = 1.92, 95% CI = 1.11–3.32) and adverse effects related to cannabis use (AOR = 4.08, 95% CI = 1.41–11.81).
(94)	Cross-sectional	1560 cannabis users	NR	High potency: ≥10% Low potency: <10%	Use of high-potency cannabis was associated with an increased risk of having psychotic experiences (AOR = 2.38, 95% CI = 1.30–4.38).
(95)	Cross-sectional	410 cannabis users (362 with potency data)	20.58 (1.66)	High potency ≥ 10% Low potency < 10%	In adjusted models, there was no impact of cannabis potency on reported symptoms of cannabis dependence, psychosis, anxiety or depression.
(96)	Cross-sectional	1309 cannabis users (655 patients with FEP, 654 controls)	Patients: 28.51 (NR) Controls: 34.30 (NR)	High potency: ≥ 10% Low potency: < 10%	Use of higher-potency cannabis was more strongly associated with an increased likelihood of having psychotic-like experiences in patients compared to controls.
(97)	Cross-sectional	2112 cannabis users (881 patients with FEP, 1231 controls)	33.97 (12.58)	Low potency: < 10% High potency: ≥ 10%	High-potency cannabis use was associated with an increased risk of developing psychosis (AOR = 1.53, 95% CI = 1.32–1.76). Cannabis potency also mediated the relationship between childhood household discord and psychosis (indirect effect coefficient 0.059, s.e. = 0.018), childhood sexual abuse and psychosis (indirect effect coefficient 0.078, s.e. = 0.029) and early childhood (≥ 12 years old) psychological abuse and psychosis (indirect effect coefficient 0.104, s.e. = 0.041).
(98)	Longitudinal (baseline and 1.5 year follow-up)	98 cannabis users	23.7 (3.01)	Mean 3.64% (range 0.42–15.72%)	Ingesting higher concentrations of THC per month was associated with an increase in CUD symptoms (*b* = 0.27, *P* = 0.03, adjusted *R* ^2^ = 0.04).

THC, tetrahydrocannabinol; NR, not reported; CUD, cannabis use disorder; HR, hazard ratio; AES-S, apathy evaluation scale; AOR, adjusted odds ratio; FEP, first-episode psychosis.

**Table 6:** Characteristics of included studies with quantitative potency and acute effects of high-potency cannabis

Primary author	Design	Sample size	Age (mean, s.d.)	Cannabinoid potency	Findings
(99)	Randomised controlled trial	25 cannabis and placebo	Median: 22.3	2.5, 5.0 or 10.0 mg THC Median dose: 320 ug/kg THC	THC administration was associated with deficits in psychomotor coordination, reaction speed and cognitive flexibility.
(100)	Non-randomised experimental study	12 adult cannabis users	25.08 (2.54)	THC: 27.39 (3.48) CBD: 0.36 (0.82)	Use of high-potency cannabis was associated with decreased ratings of anxiety. Use of high-potency cannabis had no significant impact on emotion regulation, reaction time nor inhibitory control.
(101)	Longitudinal	149 adult cannabis and alcohol users	22.04 (2.10)	Categorical self-report	Compared to alcohol and cannabis co-use, acute cannabis use was not associated with heightened anxiety in participants with higher average THC intake whereas this association was significant in participants with lower average THC intake.
(102)	Randomised controlled trial	80 cannabis users (randomised to 20 controls, 20 using THC-only cannabis, 20 using THC + CBD cannabis and 20 using concentrates)	Controls: 25.25 (7.37) THC-only: 23.50 (5.61) THC + CBD: 22.75 (4.66) Concentrates: 24.00 (4.77)	THC-only: 22.82% (3.31) THC + CBD: 22.81% (1.58) THC, 1.32% (0.51) CBD Concentrates: 71.43% (15.88) THC, 2.20% (1.60) CBD	Administration of high-potency THC-only cannabis or concentrates was associated with a decrease in self-reported stress 1 min post use and deficits in source memory compared to controls. Administration of high-potency cannabis with THC + CBD was associated with deficits in free recall compared to all other groups. Administration of any cannabis type was associated with an increased risk of developing false memories, especially after use of concentrates.
(103)	Randomised controlled trial	86 cannabis users (54 young adults, 32 older adults)	Young adults: 22.45 (1.37) Older adults: 63.13 (4.66)	CBD condition: 23% CBD, <1% THC THC condition: 24% THC, <1% CBD THC + CBD condition: 10% CBD, 9% THC	Administration of cannabis with high % THC was significantly associated with deficits in processing speed and memory. Administration of cannabis with high % THC was significantly associated with an acute increase in cannabis craving compared to the administration of high % CBD cannabis. Administration of the equal % THC and % CBD cannabis was significantly associated with an increase in cannabis craving 1 h post administration. Administration of cannabis with high % THC was significantly associated with an acute increase in anxiety compared to the administration of equal % THC and % CBD cannabis.
(104)	Randomised controlled trial	Oral study: 12 cannabis users Smoking study: 13 cannabis users	Oral study: 23.0 (4.0) Smoking study: 21.0 (2.0)	2.11% (0.06) THC, 8.4 mg of THC in low THC condition, 16.9 mg of THC in high THC condition	Cannabis potency had no significant impact on acute cognitive functioning or mood.

THC, tetrahydrocannabinol; CBD, cannabidiol.

### Long-term effects of HPC

We found that HPC use in the AYA population was most commonly associated with an increased risk and prevalence of cannabis dependence/misuse and an increased prevalence and earlier onset of psychotic disorders. Long-term HPC use was also associated with issues related to sleep quality, mood, cognition and personality, but these outcomes were less commonly reported on.

### Problematic substance use and dependence

According to the examined literature, users of HPC, particularly concentrates and high-THC percentage dried flower, exhibit higher levels of cannabis dependence and problematic cannabis use. Studies included report that, when compared with users of LPC products, users of HPC products, such as butane hash oil (BHO),^57,64^ cannabis concentrates,^58,65,77–79^ vapes^80^ or high-potency flower^59,66,67,81–85^ are at a higher risk of reporting symptoms of cannabis dependence and/or other problematic substance use. Strong evidence for this relationship is provided by Bedillion et al. Using a longitudinal rather than cross-sectional design, these authors reported that individuals who used BHO at baseline were significantly more likely to develop cannabis dependence symptoms and have experienced negative outcomes related to their cannabis use, in a dose-dependent manner.^57^ However, some studies have reported that HPC use is not associated with cannabis dependence measures^60,61,68–70,86^ or that HPC use is associated with a decrease in cannabis dependence symptoms.^69^

### Psychotic symptoms and psychotic disorders

The second most commonly examined outcome among HPC AYA studies was psychosis/psychotic symptoms. Regarding psychotic symptoms, studies included report that regular use of HPC, such as skunk (16–20% THC^105^) or cannabis concentrates is associated with having an increased number of psychotic symptoms.^59,71–73,81,87^ Regarding psychotic disorders, Di Forti et al found a strong association between HPC use and risk of psychosis. Out of a large sample of patients with first-episode psychosis (*N* = 280) and controls (*N* = 174), these authors found that daily users of skunk were significantly more likely to be a part of the patient group than those who used lower-potency cannabis products (odds ratio 12.1, 95% CI = 3.7–37.3^74^). This same relationship was observed among non-daily users of HPC as well, albeit to a lower magnitude (odds ratio 5.7, 95% CI = 2.5–12.6^74^). In later studies, Di Forti et al found that HPC use was associated with an increased risk of developing a psychotic disorder^62^ and that cannabis use of any potency was associated with an earlier onset of psychosis, with the earliest onsets being seen in those who used HPC.^88^ In individuals with already diagnosed psychotic disorders, one included study found that regular HPC use was associated with a decline in functioning.^63^ Contrary to these results, one study found that an increase in cannabis flower potency was associated with a decrease in psychotic symptoms, whereas increased concentrate potency was again associated with an increase in psychotic symptoms.^75^ Another study similarly found that HPC use was associated with a decreased risk of developing a substance-related psychotic disorder.^82^ Another reported that participants who regularly used cannabis with higher levels of cannabidiol (CBD) reported fewer psychotic symptoms than those who used HPC.^72^

### Other mental health outcomes

One study found that the potency of cannabis concentrates was positively associated with mood symptoms (anger, depression, mania and anxiety), sleep issues, personality disorder symptoms and repetitive thoughts and behaviours.^75^ However, this same study found that cannabis flower potency was *negatively* associated with memory issues, anger and repetitive thoughts and behaviours.^75^ Other studies have found that HPC use during AYA is associated with increased depressive and anxious symptoms,^59,87^ heightened aggression,^59^ increased risk of suicide,^89^ worsened cognitive ability,^76,81,90^ sleep quality degradation^71^ and deficits in occupational functioning.^59^ One of the included studies additionally compared the cognitive profile of HPC cannabis users with profiles of AYAs with known psychotic prodromes, finding them to be similar.^90^

### Acute effects of HPC use

Studies have reported that HPC administered acutely is associated with significant deficits in a number of cognitive domains including processing speed, memory, attention and executive functioning. Other less common outcomes included cannabis craving and mood.

First, Belgrave et al observed that increasing THC doses (2.5, 5.0 and 10.0 mg) significantly impaired individuals’ psychomotor coordination, reaction speed and executive functioning, with effects lasting longer at higher doses.^99^ Another study found that the administration of cannabis flower with high levels of THC and CBD led to poorer free recall and increased susceptibility to false memories compared with controls and lower-potency conditions.^102^ Additionally, cannabis concentrates produced a greater risk of developing false memories.^102^ Deficits in memory and processing speed because of increased THC concentrations were also found by another study.^103^ Regarding anxiety, findings suggest that acute HPC administration is associated with increased cannabis craving and anxiety,^103^ while other findings suggest that HPC administration is associated with reduced feelings of stress and anxiety.^100,102^ Another study reported that, compared with alcohol and HPC co-use, acute HPC intake alone was only associated with heightened anxiety among users with a lower average THC intake,^101^ indicating that the effects of HPC on anxiety might be most pronounced among individuals who are light cannabis users. Null findings regarding the impact of acute HPC use were reported by multiple studies, specifically regarding changes in cognition and mood.^100,104^

### Sex differences

The secondary aim of this review was to examine what evidence exists regarding sex and/or gender differences in the impact that cannabis potency has on AYA mental health outcomes. Unfortunately, sex and/or gender differences are highly underreported, with clear definitions of these constructs being even less common. Out of all included studies, only 14 explicitly assessed how sex could affect the relationship between cannabis potency and mental health outcomes in AYA,^34–39,58,60,73,77,78,81,88,89^ with only one reporting a significant impact of sex and/or gender on HPC and mental health outcomes. This study found that females were more susceptible to paranoid symptom development than males.^73^

## Discussion

The primary purpose of this scoping review was to summarise the existing literature regarding the impact of cannabis potency on AYA mental health outcomes. The summary provided here suggests that the use of HPC has both long-term and acute mental health effects. According to the studies reviewed here, the most common long-term effects of HPC use include: a higher risk of problematic cannabis use,^57,64,66,67^ a higher risk – and earlier onset – of psychosis and psychotic symptoms^71,72,74^ and other adverse mental health outcomes,^65,71,75^ while common acute effects of HPC include: cognitive deficits,^99,102,103,106^ increased cannabis craving,^103^ increased anxiety^103^ and decreased stress levels.^102^ However, there were a portion of studies which found that cannabis potency had no impact on mental health outcomes,^61,68,69,86,104^ which invites caution when trying to make sweeping conclusions regarding the impact of AYA HPC use on these outcomes.

The secondary aim of this review was to collate information regarding the impact that sex and/or gender has on the relationship between cannabis potency and mental health outcomes in AYA. Unfortunately, only 14 of the studies discussed here explicitly assessed sex and/or gender effects.^34–39,58,60,73,77,78,81,88,89^ Of this subset, only one found that the relationship between HPC use and AYA mental health outcomes was moderated by sex.^73^ Although these results could suggest that sex and/or gender has little impact on the relationship between cannabis potency and mental health outcomes in AYA, no strong conclusions can be made until further high-quality research on this topic is conducted. Apart from the lack of research examining sex and/or gender, what is additionally concerning is the fact that, despite having an explicit focus on sex and/or gender in their analyses, many of the aforementioned studies did not clearly define what the terms ‘sex’ and/or ‘gender’ meant in the context of their study. Delineating between these two constructs is important if we are ever to understand how someone’s sex and/or gender can impact AYA HPC use and outcomes.

The strengths of this scoping review include our search strategies, which were comprehensive and developed with the help of evidence synthesis librarians, and our use of two independent reviewers for the screening and extraction of sources. Together, we believe that these factors allow this review to provide a broad overview of existing research surrounding the effect that cannabis potency has on the mental health of AYA. However, our adherence to a scoping review methodology comes with inherent limitations. The first of which is that we did not assess the quality of included sources. While this would have provided information important for the purposes of making conclusions, such was not the goal of the current review. Our inclusion criteria were also quite lenient, introducing a great deal of heterogeneity to our results. Including assessments of sources and more strict inclusion criteria, both of which are common to systematic reviews, could have improved the interpretability and robustness of our results. However, the goal of the current review was not to *answer* the question of the effect of cannabis potency on the mental health of AYA, but to summarise the evidence that exists to address this question. Due to this aim, none of the conclusions made here are causal in nature.

Given the emerging evidence reviewed here and cannabis’ changing legal status in multiple jurisdictions, it would be suggested that researchers and governing bodies monitor product potency from a public health perspective. Currently, in Canada, the THC limit of edible cannabis is 10 mg per package while extracts can contain 1000 mg per package.^107^ While these limits provide some control, they may not be sufficient to address concerns related to high-potency products. Considering that extracts can contain significantly higher amounts of THC, stricter limits or additional controls specifically targeting younger users may be warranted. Reducing access to HPC for younger populations could lead to the decreased incidence of cannabis-related cognitive and mental health issues. However, despite these quality control measures, a recent study found that one-third of cannabis products sold in Ontario, Canada had discrepancies between online cannabinoid potency descriptions and the potency of the actual product.^108^ This study reported that 16.7% of cannabis products in Ontario had physical labels that reported higher THC potency of the advertised product.^108^ Research such as this emphasises that governing bodies should ensure that cannabis distributors are accurately reporting the THC content of their products so that potency can be properly monitored and studied.

The increasing prevalence of cannabis use among youth, along with the significant rise in cannabis potency in recent years, calls for targeted research and education regarding its effects on mental health. As cannabis products become more potent, understanding their impact on developing brains and mental well-being is critical. There is a pressing need for enhanced investment in educational programmes tailored to different age groups, focusing on the risks associated with HPC and its potential effects on mental health. Such initiatives would help equip young people with the knowledge to make informed decisions and better navigate the potential risks associated with cannabis use. Considering that AYA can access HPC outside the legal market, it is also important for governments to implement measures to limit this exposure. Synthetic cannabinoids (i.e. spice, K2) must also be the focus of future research assessing the impact of AYA cannabinoid exposure on acute- and long-term mental health outcomes

In this review, our primary aim was to collate extant literature reporting the long-term and acute mental health effects of HPC use in AYA. Included studies most commonly reported that, compared with LPC use, HPC use in AYA is associated with an increased risk of psychosis/psychotic symptoms, problematic cannabis use and cognitive deficits. Our review also noted the almost complete lack of literature on the potential of sex and/or gender to moderate the relationship between cannabis potency and mental health outcomes in AYA, as well as the ambiguity with which researchers define sex and gender. We strongly recommend that future studies assessing the impact of cannabis on mental health also consider how the potency of products might influence their findings and include sex and/or gender-based analyses into their designs.

## Supporting information

10.1192/bjo.2026.12023.sm001 Hambly Lapointe et al. supplementary material Hambly Lapointe et al. supplementary material

## Data Availability

Data availability is not applicable to this article as no new data were created or analysed in this study.

## Supplementary material

The supplementary material is available online at https://doi.org/10.1192/bjo.2026.12023

## Appendix A

**MEDLINE (Ovid)** – 3 June 2024

1. adolescent/ OR ‘young adult’/ OR (teen* OR youth* OR adolescen* OR juvenile* OR (young ADJ2 (adult* OR person* OR individual* OR people* OR population* OR man OR men OR wom?n)) OR youngster* OR highschool* OR college* OR ((secondary OR high*) ADJ2 (school* OR education))).kf,ti,ab.
2. exp cannabinoids/ OR exp cannabis/ OR exp ‘marijuana abuse’/ OR exp ‘marijuana use’/ OR exp ‘medical marijuana’/ OR (cannabi* OR dronabinol OR hash* OR mari?uana* OR tetrahydrocannabi* OR THC OR hemp* OR bhang*).kf,ti,ab.
3. (strength OR strong OR concentrat* OR mass OR potency OR potent OR ‘%THC’ OR ‘% THC’ OR percent* OR ‘mg THC’ OR ‘mgTHC’ OR ‘THC:CBD ratio’ OR quanti* OR content OR milligram* OR ‘mg’).kf,ti,ab.
4. exp mental disorders/ OR ((psych* OR mental OR cogniti* OR panic OR eating OR mood OR ‘substance use’ OR conduct OR personality OR ‘attention?deficit?hyperactive’ OR ‘Obsessive?Compulsive’) ADJ1 (ill* OR disord* OR condition* OR disab*) OR anx* OR depres* OR schizo* OR bipolar OR suicid* OR anorex* OR agoraphob* OR PTSD OR post?traumatic?stress* OR bulimi* OR ADHD OR OCD OR delusions OR hallucinations OR ‘substance abuse’ OR ‘substance misuse’ OR phobia OR ‘panic attack*’ OR ‘gender dysphori*’).kf,ti,ab.
5. 1 AND 2 AND 3 AND 4
6. exp Animals/ NOT (exp Animals/AND Humans/)
7. (news OR comment OR editorial OR interview OR letter OR proceedings).pt.
8. 5 NOT 6 NOT 7

**MEDLINE (Ovid)** – 3 October 2025

1. adolescent/ OR ‘young adult’/ OR (teen* OR youth* OR adolescen* OR juvenile* OR (young ADJ2 (adult* OR person* OR individual* OR people* OR population* OR man OR men OR wom?n)) OR youngster* OR highschool* OR college* OR ((secondary OR high*) ADJ2 (school* OR education))).kf,ti,ab.
2. exp cannabinoids/ OR exp cannabis/ OR exp ‘marijuana abuse’/ OR exp ‘marijuana use’/ OR exp ‘medical marijuana’/ OR (cannabi* OR dronabinol OR hash* OR mari?uana* OR tetrahydrocannabi* OR THC OR hemp* OR bhang*).kf,ti,ab.
3. (strength OR strong OR concentrat* OR mass OR potency OR potent OR ‘%THC’ OR ‘% THC’ OR percent* OR ‘mg THC’ OR ‘mgTHC’ OR ‘THC:CBD ratio’ OR quanti* OR content OR milligram* OR ‘mg’).kf,ti,ab.
4. exp mental disorders/ OR ((psych* OR mental OR cogniti* OR panic OR eating OR mood OR ‘substance use’ OR conduct OR personality OR ‘attention?deficit?hyperactive’ OR ‘Obsessive?Compulsive’) ADJ1 (ill* OR disord* OR condition* OR disab*) OR anx* OR depres* OR schizo* OR bipolar OR suicid* OR anorex* OR agoraphob* OR PTSD OR post?traumatic?stress* OR bulimi* OR ADHD OR OCD OR delusions OR hallucinations OR ‘substance abuse’ OR ‘substance misuse’ OR phobia OR ‘panic attack*’ OR ‘gender dysphori*’).kf,ti,ab.
5. 1 AND 2 AND 3 AND 4
6. exp Animals/ NOT (exp Animals/ AND Humans/)
7. (news OR comment OR editorial OR interview OR letter OR proceedings).pt.
8. 5 NOT 6 NOT 7
9. limit 8 to yr=’2024 -Current’

**Embase (Elsevier)** – 3 June 2024

1. ‘adolescent’/exp OR ‘young adult’/exp OR (teen* OR youth* OR adolescen* OR juvenile* OR (young NEAR/1 (adult* OR person* OR individual* OR people* OR population* OR man OR men OR wom?n)) OR youngster* OR highschool* OR college* OR ((secondary OR high*) NEAR/1(school* OR education))):kw,ti,ab
2. ‘cannabis’/exp OR ‘cannabinoid’/exp OR ‘cannabis use’/exp OR ‘cannabis addiction’/exp OR ‘cannabis sativa’/exp OR ‘medical cannabis’/exp OR (cannabi* OR dronabinol OR hash* OR mari?uana* OR tetrahydrocannabi* OR THC OR hemp* OR bhang*):kw,ti,ab
3. (strength OR strong OR concentrat* OR mass OR potency OR potent OR ‘%THC’ OR ‘% THC’ OR percent* OR ‘mg THC’ OR ‘mgTHC’ OR ‘THC:CBD ratio’ OR quanti* OR content OR milligram* OR ‘mg’):kw,ti,ab
4. ‘mental disease’/exp OR ((mental OR psych* OR cogniti* OR panic OR eating OR mood OR conduct OR personality OR ‘attention?deficit?hyperactive’ OR ‘substance use’ OR ‘Obsessive?Compulsive’) NEAR/1 (ill* OR disord* OR condition* OR disab*) OR bipolar OR anx* or depres* OR schizo* OR suicid* OR anorex* OR agoraphob* OR PTSD OR post?traumatic?stress* OR bulimi* OR ADHD OR OCD OR delusions OR hallucinations OR phobia OR ‘substance abuse’ OR ‘substance misuse’ OR ‘panic attack*’ OR ‘gender dysphor*’):kw,ti,ab
5. 1 AND 2 AND 3 AND 4
6. (news OR comment OR editorial OR interview OR letter OR proceedings):it
7. ‘animal experiment’/de NOT (‘human experiment’/de OR ‘human’/de)
8. 5 NOT 6 NOT 7

**Embase (Elsevier)** – 3 October 2025

1. ‘adolescent’/exp OR ‘young adult’/exp OR (teen* OR youth* OR adolescen* OR juvenile* OR (young NEAR/1 (adult* OR person* OR individual* OR people* OR population* OR man OR men OR wom?n)) OR youngster* OR highschool* OR college* OR ((secondary OR high*) NEAR/1(school* OR education))):kw,ti,ab
2. ‘cannabis’/exp OR ‘cannabinoid’/exp OR ‘cannabis use’/exp OR ‘cannabis addiction’/exp OR ‘cannabis sativa’/exp OR ‘medical cannabis’/exp OR (cannabi* OR dronabinol OR hash* OR mari?uana* OR tetrahydrocannabi* OR THC OR hemp* OR bhang*):kw,ti,ab
3. (strength OR strong OR concentrat* OR mass OR potency OR potent OR ‘%THC’ OR ‘% THC’ OR percent* OR ‘mg THC’ OR ‘mgTHC’ OR ‘THC:CBD ratio’ OR quanti* OR content OR milligram* OR ‘mg’):kw,ti,ab
4. ‘mental disease’/exp OR ((mental OR psych* OR cogniti* OR panic OR eating OR mood OR conduct OR personality OR ‘attention?deficit?hyperactive’ OR ‘substance use’ OR ‘Obsessive?Compulsive’) NEAR/1 (ill* OR disord* OR condition* OR disab*) OR bipolar OR anx* or depres* OR schizo* OR suicid* OR anorex* OR agoraphob* OR PTSD OR post?traumatic?stress* OR bulimi* OR ADHD OR OCD OR delusions OR hallucinations OR phobia OR ‘substance abuse’ OR ‘substance misuse’ OR ‘panic attack*’ OR ‘gender dysphor*’):kw,ti,ab
5. 1 AND 2 AND 3 AND 4
6. (news OR comment OR editorial OR interview OR letter OR proceedings):it
7. ‘animal experiment’/de NOT (‘human experiment’/de OR ‘human’/de)
8. 5 NOT 6 NOT 7
9. #8 AND (**2024**:py OR **2025**:py)

**CINAHL (EBSCO)** – 3 June 2024

1. (MH ‘Adolescence’) OR (MH ‘Young Adult’) OR TI (teen* OR youth* OR adolescen* OR juvenile* OR (young N1(adult* OR person* OR individual* OR people* OR population* OR man OR men OR wom?n)) OR youngster* OR highschool* OR college* OR ((secondary OR high*) N1 (school* OR education))) OR AB (teen* OR youth* OR adolescen* OR juvenile* OR (young N1 (adult* OR person* OR individual* OR people* OR population* OR man OR men OR wom?n)) OR youngster* OR highschool* OR college* OR ((secondary OR high*) N1 (school* OR education)))
2. (MH ‘Medical Marijuana’) OR (MH ‘Cannabidiol’) OR (MH ‘Cannabis+’) OR TI (cannabi* OR dronabinol OR hash* OR mari?uana* OR tetrahydrocannabi* OR THCOR hemp* OR bhang*) OR AB (cannabi* OR dronabinol OR hash* OR mari?uana* OR tetrahydrocannabi* OR THCOR hemp* OR bhang*)
3. TI (strength OR strong OR concentrat* OR mass OR potency OR potent OR ‘%THC’ OR ‘% THC’ OR percent* OR ‘mg THC’ OR ‘mgTHC’ OR ‘THC:CBD ratio’ OR quanti* OR content OR milligram* OR ‘mg’) OR AB (strength OR strong OR concentrat* OR mass OR potency OR potent OR ‘%THC’ OR ‘% THC’ OR percent* OR ‘mg THC’ OR ‘mgTHC’ OR ‘THC:CBD ratio’ OR quanti* OR content OR milligram* OR ‘mg’)
4. (MH ‘Behavioral and Mental Disorders+’) OR TI ((psych* OR mental OR cogniti* OR panic OR eating OR mood OR ‘substance use’ OR conduct OR personality OR ‘attention?deficit?hyperactive’ OR ‘Obsessive?Compulsive’) ADJ1 (ill* OR disord* OR condition* OR disab*) OR anx* OR depres* OR schizo* OR bipolar OR suicid* OR anorex* OR agoraphob* OR PTSD OR post?traumatic?stress* OR bulimi* OR ADHD OR OCD OR delusions OR hallucinations OR ‘substance abuse’ OR ‘substance misuse’ OR phobia OR ‘panic attack*’ OR ‘gender dysphori*’) OR AB ((psych* OR mental OR cogniti* OR panic OR eating OR mood OR ‘substance use’ OR conduct OR personality OR ‘attention?deficit?hyperactive’ OR ‘Obsessive?Compulsive’) ADJ1 (ill* OR disord* OR condition*OR disab*) OR anx* OR depres* OR schizo* OR bipolar OR suicid* OR anorex* OR agoraphob* OR PTSD OR post?traumatic?stress* OR bulimi* OR ADHD OR OCD OR delusions OR hallucinations OR ‘substance abuse’ OR ‘substance misuse’ OR phobia OR ‘panic attack*’ OR ‘gender dysphori*’)
5. 1 AND 2 AND 3 AND 4
6. PT (news OR comment OR editorial OR interview OR letter OR proceedings)
7. TI ((animal OR animals OR canine* OR dog OR dogs OR feline OR hamster* OR lamb OR lambs OR mice OR monkey OR monkeys OR mouse OR murine OR pig OR pigs OR piglet* OR porcine OR primate* OR rabbit* OR rats OR rat OR rodent* OR sheep*) NOT (human* OR patient*))
8. AB ((animal OR animals OR canine* OR dog OR dogs OR feline OR hamster* OR lamb OR lambs OR mice OR monkey OR monkeys OR mouse OR murine OR pig OR pigs OR piglet* OR porcine OR primate* OR rabbit* OR rats OR rat OR rodent* OR sheep*) NOT (human* OR patient*))
9. 5 NOT 6 NOT 7 NOT 8

**CINAHL (EBSCO)** – 3 October 2025

1. (MH ‘Adolescence’) OR (MH ‘Young Adult’) OR TI (teen* OR youth* OR adolescen* OR juvenile* OR (young N1(adult* OR person* OR individual* OR people* OR population* OR man OR men OR wom?n)) OR youngster* OR highschool* OR college* OR ((secondary OR high*) N1 (school* OR education))) OR AB (teen* OR youth* OR adolescen* OR juvenile* OR (young N1 (adult* OR person* OR individual* OR people* OR population* OR man OR men OR wom?n)) OR youngster* OR highschool* OR college* OR ((secondary OR high*) N1 (school* OR education)))
2. (MH ‘Medical Marijuana’) OR (MH ‘Cannabidiol’) OR (MH ‘Cannabis+’) OR TI (cannabi* OR dronabinol OR hash* OR mari?uana* OR tetrahydrocannabi* OR THCOR hemp* OR bhang*) OR AB (cannabi* OR dronabinol OR hash* OR mari?uana* OR tetrahydrocannabi* OR THCOR hemp* OR bhang*)
3. TI (strength OR strong OR concentrat* OR mass OR potency OR potent OR ‘%THC’ OR ‘% THC’ OR percent* OR ‘mg THC’ OR ‘mgTHC’ OR ‘THC:CBD ratio’ OR quanti* OR content OR milligram* OR ‘mg’) OR AB (strength OR strong OR concentrat* OR mass OR potency OR potent OR ‘%THC’ OR ‘% THC’ OR percent* OR ‘mg THC’ OR ‘mgTHC’ OR ‘THC:CBD ratio’ OR quanti* OR content OR milligram* OR ‘mg’)
4. (MH ‘Behavioral and Mental Disorders+’) OR TI ((psych* OR mental OR cogniti* OR panic OR eating OR mood OR ‘substance use’ OR conduct OR personality OR ‘attention?deficit?hyperactive’ OR ‘Obsessive?Compulsive’) ADJ1 (ill* OR disord* OR condition* OR disab*) OR anx* OR depres* OR schizo* OR bipolar OR suicid* OR anorex* OR agoraphob* OR PTSD OR post?traumatic?stress* OR bulimi* OR ADHD OR OCD OR delusions OR hallucinations OR ‘substance abuse’ OR ‘substance misuse’ OR phobia OR ‘panic attack*’ OR ‘gender dysphori*’) OR AB ((psych* OR mental OR cogniti* OR panic OR eating OR mood OR ‘substance use’ OR conduct OR personality OR ‘attention?deficit?hyperactive’ OR ‘Obsessive?Compulsive’) ADJ1 (ill* OR disord* OR condition*OR disab*) OR anx* OR depres* OR schizo* OR bipolar OR suicid* OR anorex* OR agoraphob* OR PTSD OR post?traumatic?stress* OR bulimi* OR ADHD OR OCD OR delusions OR hallucinations OR ‘substance abuse’ OR ‘substance misuse’ OR phobia OR ‘panic attack*’ OR ‘gender dysphori*’)
5. 1 AND 2 AND 3 AND 4
6. PT (news OR comment OR editorial OR interview OR letter OR proceedings)
7. TI ((animal OR animals OR canine* OR dog OR dogs OR feline OR hamster* OR lamb OR lambs OR mice OR monkey OR monkeys OR mouse OR murine OR pig OR pigs OR piglet* OR porcine OR primate* OR rabbit* OR rats OR rat OR rodent* OR sheep*) NOT (human* OR patient*))
8. AB ((animal OR animals OR canine* OR dog OR dogs OR feline OR hamster* OR lamb OR lambs OR mice OR monkey OR monkeys OR mouse OR murine OR pig OR pigs OR piglet* OR porcine OR primate* OR rabbit* OR rats OR rat OR rodent* OR sheep*) NOT (human* OR patient*))
9. 5 NOT 6 NOT 7 NOT 8
10. Limited publication date to: 20240601-20251231

**PsycINFO (EBSCO)** – 3 June 2024

1. DE ‘Emerging Adulthood’ OR DE ‘Adolescent Development’ OR DE ‘Adolescent Behavior’ OR DE ‘Adolescent Characteristics’ OR DE ‘Adolescent Health’ OR DE ‘Late Adolescence’ OR DE ‘Youth Mental Health’ OR TI ((teen* OR youth* OR adolescen* OR juvenile* OR (young N2 (adult* OR person* OR individual* OR people* OR population* OR man OR men OR wom?n)) OR youngster* OR highschool* OR college* OR ((secondary OR high*) N2 (school* OR education)))) OR AB ((teen* OR youth* OR adolescen* OR juvenile* OR (young N2 (adult* OR person* OR individual* OR people* OR population* OR man OR men OR wom?n)) OR youngster* OR highschool* OR college* OR ((secondary OR high*) N2 (school* OR education))))
2. DE ‘Cannabis’ OR DE ‘Cannabinoids’ OR DE ‘Hashish’ OR DE ‘Marijuana’ OR DE ‘Medical Marijuana’ OR TI (cannabi* OR dronabinol OR hash* OR mari?uana* OR tetrahydrocannabi* OR THC OR hemp* OR bhang*) OR AB (cannabi* OR dronabinol OR hash* OR mari?uana* OR tetrahydrocannabi* OR THC OR hemp* OR bhang*)
3. TI (strength OR strong OR concentrat* OR mass OR potency OR potent OR ‘%THC’ OR ‘% THC’ OR percent* OR ‘mg THC’ OR ‘mgTHC’ OR ‘THC:CBD ratio’ OR quanti* OR content OR milligram* OR ‘mg’) OR AB (strength OR strong OR concentrat* OR mass OR potency OR potent OR ‘%THC’ OR ‘% THC’ OR percent* OR ‘mg THC’ OR ‘mgTHC’ OR ‘THC:CBD ratio’ OR quanti* OR content OR milligram* OR ‘mg’)
4. DE ‘Mental Disorders’ OR DE ‘Affective Disorders’ OR DE ‘Anxiety Disorders’ OR DE ‘Behavior Disorders’ OR DE ‘Bipolar Disorder’ OR DE ‘Borderline States’ OR DE ‘Chronic Mental Illness’ OR DE ‘Dissociative Disorders’ OR DE ‘Eating Disorders’ OR DE ‘Gender Dysphoria’ OR DE ‘Mental Disorders due to General Medical Conditions’ OR DE ‘Neurocognitive Disorders’ OR DE ‘Neurodevelopmental Disorders’ OR DE ‘Neurosis’ OR DE ‘Obsessive Compulsive Disorder’ OR DE ‘Paraphilias’ OR DE ‘Personality Disorders’ OR DE ‘Psychosis’ OR DE ‘Serious Mental Illness’ OR DE ‘Sleep Wake Disorders’ OR DE ‘Somatoform Disorders’ OR DE ‘Stress and Trauma Related Disorders’ OR DE ‘Substance Related and Addictive Disorders’ OR DE ‘Thought Disorders’ OR DE ‘Youth Suicide’ OR DE ‘Abnormal Psychology’ OR DE ‘Attention Deficit Disorder’ OR DE ‘Attention Deficit Disorder with Hyperactivity’ OR DE ‘Suicide’ OR TI ((psych* OR mental OR cogniti* OR panic OR eating OR mood OR ‘substance use’ OR conduct OR personality OR ‘attention?deficit?hyperactive’ OR ‘Obsessive?Compulsive’) ADJ1 (ill* OR disord* OR condition* OR disab*) OR anx* OR depres* OR schizo* OR bipolar OR suicid* OR anorex* OR agoraphob* OR PTSD OR post?traumatic?stress* OR bulimi* OR ADHD OR OCD OR delusions OR hallucinations OR ‘substance abuse’ OR ‘substance misuse’ OR phobia OR ‘panic attack*’ OR ‘gender dysphori*’) OR AB ((psych* OR mental OR cogniti* OR panic OR eating OR mood OR ‘substance use’ OR conduct OR personality OR ‘attention?deficit?hyperactive’ OR ‘Obsessive?Compulsive’) ADJ1 (ill* OR disord* OR condition* OR disab*) OR anx* OR depres* OR schizo* OR bipolar OR suicid* OR anorex* OR agoraphob* OR PTSD OR post?traumatic?stress* OR bulimi* OR ADHD OR OCD OR delusions OR hallucinations OR ‘substance abuse’ OR ‘substance misuse’ OR phobia OR ‘panic attack*’ OR ‘gender dysphori*’)
5. 1 AND 2 AND 3 AND 4
6. PT (news OR comment OR editorial OR interview OR letter OR proceedings)
7. TI ((animal OR animals OR canine* OR dog OR dogs OR feline OR hamster* OR lamb OR lambs OR mice OR monkey OR monkeys OR mouse OR murine OR pig OR pigs OR piglet* OR porcine OR primate* OR rabbit* OR rats OR rat OR rodent* OR sheep*) NOT (human* OR patient*))
8. AB ((animal OR animals OR canine* OR dog OR dogs OR feline OR hamster* OR lamb OR lambs OR mice OR monkey OR monkeys OR mouse OR murine OR pig OR pigs OR piglet* OR porcine OR primate* OR rabbit* OR rats OR rat OR rodent* OR sheep*) NOT (human* OR patient*))
9. 5 NOT 6 NOT 7 NOT 8

**PsycINFO (Ovid)** – 3 October 2025

1. exp Adolescent Behavior/ or exp Adolescent Development/ or exp Adolescent Characteristics/ or exp Adolescent Health/ OR exp Emerging Adulthood/ OR exp Late Adolescence/ OR exp Youth Mental Health/ OR (teen* OR youth* OR adolescen* OR juvenile* OR (young ADJ2 (adult* OR person* OR individual* OR people* OR population* OR man OR men OR wom?n)) OR youngster* OR highschool* OR college* OR ((secondary OR high*) ADJ2 (school* OR education))).hw,ti,ab.
2. exp Cannabis/ OR exp Cannabinoids/ OR exp Hashish/ OR exp Marijuana/ OR exp Medical Marijuana/ OR (cannabi* OR dronabinol OR hash* OR mari?uana* OR tetrahydrocannabi* OR THC OR hemp* OR bhang*).hw,ti,ab.
3. (strength OR strong OR concentrat* OR mass OR potency OR potent OR ‘%THC’ OR ‘% THC’ OR percent* OR ‘mg THC’ OR ‘mgTHC’ OR ‘THC:CBD ratio’ OR quanti* OR content OR milligram* OR ‘mg’).hw,ti,ab.
4. exp Mental Disorders/ OR exp Affective Disorders/ OR exp Anxiety Disorders/ OR exp Behavior Disorders/ OR exp Bipolar Disorder/ OR exp Borderline States/ OR exp Chronic Mental Illness/ OR exp Dissociative Disorders/ OR exp Eating Disorders/ OR exp Gender Dysphoria/ OR exp Mental Disorders due to General Medical Conditions/ OR exp Neurocognitive Disorders/ OR exp Neurodevelopmental Disorders/ OR exp Neurosis/ OR exp Obsessive Compulsive Disorder/ OR exp Paraphilias/ OR exp Personality Disorders/ OR exp Psychosis/ OR exp Serious Mental Illness/ OR exp Sleep Wake Disorders/ OR exp Somatoform Disorders/ OR exp Stress and Trauma Related Disorders/ OR exp Substance Related and Addictive Disorders/ OR exp Thought Disorders/ OR exp Youth Suicide/ OR exp Abnormal Psychology/ OR exp Attention Deficit Disorder/ OR exp Attention Deficit Disorder with Hyperactivity/ OR exp Suicide/ OR ((psych* OR mental OR cogniti* OR panic OR eating OR mood OR ‘substance use’ OR conduct OR personality OR ‘attention?deficit?hyperactive’ OR ‘Obsessive?Compulsive’) ADJ1 (ill* OR disord* OR condition* OR disab*) OR anx* OR depres* OR schizo* OR bipolar OR suicid* OR anorex* OR agoraphob* OR PTSD OR post?traumatic?stress* OR bulimi* OR ADHD OR OCD OR delusions OR hallucinations OR ‘substance abuse’ OR ‘substance misuse’ OR phobia OR ‘panic attack*’ OR ‘gender dysphori*’).hw,ti,ab.
5. 1 AND 2 AND 3 AND 4
6. exp Animals/ NOT (exp Animals/ AND Humans/)
7. (news OR comment OR editorial OR interview OR letter OR proceedings).pt.
8. 5 NOT 6 NOT 7
9. limit 8 to yr=‘2024 -Current’

## Fig. 1 Long description

The flowchart illustrates the process of screening and selecting studies for a review on cannabis. It begins with the identification phase, where studies from databases/ registers are listed, totaling 11225 studies. These databases include Embase (4547), MEDLINE (3214), CINAHL (1577), and PsycINFO (724). Next, references removed are shown, totaling 3681, with duplicates identified manually (63) and by Covidence (3618). The screening phase follows, where 7544 studies are screened, and 6771 studies are excluded. Studies sought for retrieval total 773, with 25 studies not retrieved. Studies assessed for eligibility total 748, with 677 studies excluded for various reasons such as review (56), no potency (156), ineligible study design (106), ineligible cannabis type (184), ineligible population age (128), ineligible population animals (4), ineligible/no mental health outcomes (29), and no English translation available (14). Finally, 71 studies are included in the review.

Navigate back to Fig. 1.

## Table 4 Long description

The table presents a detailed summary of studies focusing on the long-term effects of high potency cannabis. It includes 14 studies with a cross-sectional design and qualitative measures of potency. The table has 14 rows and 6 columns. Column headers are: Ref, Study design, Sample, Age (mean (s.d.), range), Cannabis potency, and Findings. Row 1: Ref: 37, Study design: Cross-sectional, Sample: 1390 cannabis-using college students, Age: 19.84 (1.34, 18-24), Cannabis potency: Categorical self-report, Findings: Primarily concentrate cannabis users had significantly higher scores on the CUDIT-R but endorsed similar cannabis consequences as the class of primarily flower users. Row 2: Ref: 38, Study design: Cross-sectional, Sample: 695 adult cannabis users (474 flower, 139 concentrate, 82 edible), Age: Flower users: 27.22 (6.04), Concentrate: 25.97 (4.92), Edible users: 27.43 (7.55), Cannabis potency: Categorical self-report, Findings: Use of high potency cannabis use was significantly associated with increased problematic cannabis use. Row 3: Ref: 39, Study design: Cross-sectional, Sample: 387 cannabis users (230 flower users, 157 concentrate users), Age: Flower users: 19.1 (1.6), Concentrate users: 18.4 (0.8), Cannabis potency: Categorical self-report, Findings: Use of high potency cannabis was associated with a greater prevalence of negative outcomes related to cannabis use (e.g., blackouts, social/interpersonal consequences, risky behavior, diminished self-perception, lack of self-care, academic/occupational consequences, impaired control and dependence symptoms). Row 4: Ref: 40, Study design: Cross-sectional, Sample: 69 adolescent cannabis users, Age: 16.4 (12-18), Cannabis potency: Categorical self-report, Findings: High potency cannabis use was associated with an increased likelihood of CUD diagnosis. Row 5: Ref: 41, Study design: Cross-sectional, Sample: 2514 cannabis users, Age: 24.25 (6.86), Cannabis potency: Categorical self-report, Findings: Use of high potency cannabis was associated with an increased number of cannabis dependence symptoms and with experiences of paranoia and delusions in memory. Row 6: Ref: 42, Study design: Cross-sectional, Sample: 71 cannabis users with psychiatric conditions, Age: 35.1 (10.2), Cannabis potency: Categorical self-report, Findings: Use of high potency cannabis was associated with an increased risk of developing a cannabis dependence disorder and a decreased risk of developing a substance-use-related psychotic disorder. Row 7: Ref: 43, Study design: Cross-sectional, Sample: 74 cannabis users, Age: Median (MAD): 21 (2.0), Cannabis potency: Ordinal and categorical self-report, Findings: Use of higher potency cannabis was associated with an increased number of cannabis dependence symptoms endorsed and an increased prevalence of withdrawal and craving. Row 8: Ref: 44, Study design: Cross-sectional, Sample: 1007 cannabis users, Age: 19.2 (0.8), Cannabis potency: Categorical self-report, Findings: Use of higher potency forms of cannabis was associated with an increased risk of experiencing an increase in cannabis dependence symptoms. Row 9: Ref: 45, Study design: Cross-sectional, Sample: 1412 students who use cannabis, Age: THC only: 19.69 (2.55), CBD only: 20.31 (3.12), THC + CBD: 20.36 (3.01), Non-users: 19.20 (2.56), Cannabis potency: Categorical self-report, Findings: Compared to using cannabis with little or no THC, use of cannabis with a higher THC content was associated with increased polysubstance use, problematic alcohol use, past-year binge drinking and past-month tobacco, alcohol and e-cigarette use. Compared to CBD and THC co-use, THC-only cannabis was associated with a lower age of tobacco, alcohol and THC use onset. Row 10: Ref: 46, Study design: Cross-sectional, Sample: 4669 students (2485 cannabis), Age: 19.62 (2.05), Cannabis potency: Categorical self-report, Findings: Cannabis potency was not associated with any mental health outcome. Row 11: Ref: 47, Study design: Cross-sectional, Sample: 209 adolescent users (low potency: 38, high potency: 171), Age: Low potency: 13.75 (0.37), High potency: 13.76 (0.39), Control: 13.69 (0.40), Cannabis potency: Categorical self-report, Findings: Compared to cannabis users, use of high potency cannabis was associated with an increased likelihood of reporting a probable anxiety disorder (AOR = 1.45, 95 percent CI: 0.96-2.20), depressive disorder (AOR = 1.59, 95 percent CI: 1.06-2.39), and auditory hallucinations (AOR = 1.56, 95 percent CI: 0.98-2.47). Row 12: Ref: 48, Study design: Cross-sectional, Sample: 410 FEP (256 users, 154 non-users), Age: Users: 28.2 (8.0), Non-users: 31.4 (9.9), Cannabis potency: Categorical self-report, Findings: Cannabis potency of any potency was associated with an earlier onset of psychosis, with regular use of high potency cannabis being associated with the earliest age of onset. Row 13: Ref: 49, Study design: Cross-sectional, Sample: 29 cannabis users, Age: 20.86 (3.71), Cannabis potency: Self-report, Findings: Use of high potency cannabis was associated with worsened verbal fluency and verbal memory and cognitive symptoms similar to those with prodromal psychotic disorders.

Navigate back to Table 4.

## Table 5 Long description

The table presents data from studies on the long-term effects of low potency cannabis. It includes columns for author (year), study design, sample size, age (mean, standard deviation, range), cannabis potency (percent THC), and findings. The table has 8 rows and 6 columns. Row 1: Author (year): 85; Study design: Longitudinal (time points: 3-year intervals ages 12-14 to ages 24-26; annual assessments ages 11-26); Sample size: 527 cannabis users; Age: NR; Cannabis potency (% THC): National average of 3.50 percent in 1994 to 12.30 percent in 2012; Findings: As the national average cannabis potency increased, the risk of new users transitioning to experience CUD symptoms increased (HR = 1.36, 95% CI = 1.20-1.54) when controlling for daily use, birth year, and sex. When controlling for regular use instead of daily use, this risk of later developing CUD symptoms increased (HR = 1.41, 95% CI = 1.24-1.60). Row 2: Author (year): 86; Study design: Cross sectional study; Sample size: 14 cannabis users; Age: 20.4 (1.3); Cannabis potency (% THC): 8.6 percent (4.0); Findings: All participant scores on the AES-S were greater than the general population mean. Use of high-potency cannabis was associated with an increased risk of experiencing generalized anxiety disorder (AOR = 1.92, 95% CI = 1.11-3.32) and adverse effects related to cannabis use (AOR = 4.08, 95% CI = 1.41-11.81). Row 3: Author (year): 87; Study design: Cross-sectional; Sample size: 1087 cannabis users; Age: NR; Cannabis potency (% THC): High potency: ≥ 10 percent, Low potency: < 10 percent; Findings: Use of high-potency cannabis was associated with an increased risk of having psychotic experiences (AOR = 2.38, 95% CI = 1.30-4.38). Row 4: Author (year): 88; Study design: Cross-sectional; Sample size: 1560 cannabis users; Age: NR; Cannabis potency (% THC): High potency: ≥ 10 percent, Low potency: < 10 percent; Findings: In adjusted models, there was no impact of cannabis potency on reported symptoms of cannabis dependence, psychosis, anxiety, or depression. Row 5: Author (year): 89; Study design: Cross-sectional; Sample size: 410 cannabis users (362 with potency data); Age: 20.58 (1.66); Cannabis potency (% THC): High potency: ≥ 10 percent, Low potency: < 10 percent; Findings: Use of higher potency cannabis was more strongly associated with an increased likelihood of having psychotic-like experiences in patients compared to controls. Row 6: Author (year): 90; Study design: Cross-sectional; Sample size: 1309 cannabis users (655 patients with FEP, 654 controls); Age: Patients: 28.51 (NR), Controls: 34.30 (NR); Cannabis potency (% THC): High potency: >= 10 percent, Low potency: < 10 percent; Findings: High potency cannabis use was associated with an increased risk of developing psychosis (AOR = 1.53, 95% CI = 1.32-1.76). Cannabis potency also mediated the relationship between childhood household discord and psychosis (indirect effect coefficient 0.059, s.e. = 0.018), childhood sexual abuse and psychosis (indirect effect coefficient 0.078, s.e. = 0.029) and childhood (age < 12 years old) psychological abuse and psychosis (indirect effect coefficient 0.104, s.e. = 0.041). Row 7: Author (year): 91; Study design: Cross-sectional; Sample size: 2112 cannabis users (881 patients with FEP, 1231 controls); Age: 33.97 (12.58); Cannabis potency (% THC): Low potency: < 10 percent, High potency: ≥ 10 percent; Findings: High potency cannabis use was associated with an increased risk of developing psychosis (AOR = 1.53, 95% CI = 1.32-1.76). Cannabis potency also mediated the relationship between childhood household discord and psychosis (indirect effect coefficient 0.059, s.e. = 0.018), childhood sexual abuse and psychosis (indirect effect coefficient 0.078, s.e. = 0.029) and childhood (age < 12 years old) psychological abuse and psychosis (indirect effect coefficient 0.104, s.e. = 0.041). Row 8: Author (year): 92; Study design: Longitudinal (Baseline and 1.5 year follow-up); Sample size: 98 cannabis users; Age: 23.7 (3.01); Cannabis potency (% THC): Mean 3.64 percent (range 0.42-15.72 percent); Findings: Ingesting higher concentrations of THC per month was associated with an increase in CUD symptoms (b = 0.27, P = 0.03, adjusted R^2 = 0.04).

Navigate back to Table 5.
