# Supplementary material for: Influence of cannabis potency on mental health outcomes among adolescents and young adults: scoping review
Source: BJPsych Open. 2026 Jul 7;12(4):e177. doi: 10.1192/bjo.2026.12023 (PMC13359059; doi:10.1192/bjo.2026.12023)
Supplement: Hambly Lapointe et al. supplementary material [file S2056472426120237sup001.docx]

**Table S1.** Characteristics of included studies with quantitative potency and acute effects of low potency cannabis

| **Author(s)** | **Study Design** | **Sample** | **Age (Mean (s.d.), Range)** | **Cannabis Potency (%THC)** | **Findings** |
| --- | --- | --- | --- | --- | --- |
| (76) | Randomized controlled trial | 10 cannabis users | 24.5 (NR) | 0.0001%, 1.77% and 3.58% THC | Administration of the 3.58% THC cannabis was associated with an increased likelihood of risky decision-making compared to placebo. |
| (77) | Randomized controlled trial | 28 cannabis users | Males: 22.6 (s.e. = 0.7)  Females: 22.5 (s.e. = 0.7) | 12.5% (2.0) THC | Cannabis administration was not associated with any changes in cognitive performance.  No sex differences in the acute effects of the administrated cannabis were observed. |
| (78) | Randomized controlled trial | 16 cannabis users | 23.6 (2.9) | 2.2% THC | Administration of cannabis coincidentally with alcohol was associated with a decreased visual search frequency while driving. |
| (79) | Randomized controlled trial | 136 cannabis users (34 in the TTRT, TPRT, TTRP, TPRP) | TTRT: 21.3 (3.5)  TPRT: 21.3 (3.7)  TTRP: 21.8 (2.8)  TPRP: 21.2 (2.4) | 0% or 2.8 % THC | Administration of the 2.8% THC cannabis was associated with decreased accuracy (B = −1.28, s.e. = .57, *sr*^2^ = .02, p = .03) and increased RT (B = 19.76, s.e. = 9.72, *sr*^2^ = .03, p = .04) on incongruent trials of the Stroop Color-Word task. |
| (80) | Randomized controlled trial | 73 cannabis users (25 males and 15 females in placebo group, 24 males and 9 females in THC group) | Placebo males: 19.8 (2.1)  Placebo females: 21.0 (2.6)  THC males: 20.2 (2.6)  THC females: 21.4 (3.6) | 0% or 2.9% THC,  22.9 mg of THC per active cigarette | No significant differences in cognitive performance between the cannabis and placebo groups were observed. |
| (81) | Randomized controlled trial | 89 cannabis users | 21.4 (4.5) | 2.7 - 3.0% THC | Administration of cannabis was associated with increased RT when responding to neutral images negatively valanced images, and cannabis-related images. |
| (93) | Randomized controlled trial | 9 females with anorexia nervosa | 45.0 (3.2) | 1-2 mg of THC/day | Among the 5 patients aged between 14 and 25, 3 gained weight over the test period while 2 lost weight. |
| (94) | Cross sectional study | 19 cannabis users with PLEs and 19 nonusers | Users: 20.8 (1.7)  Nonusers: 22.3 (2.8) | 8.7% (3.8) THC | Cannabis administration was associated with a mean increase of 9.9 (5.1) points on the Psychotomimetic States Inventory psychotic symptom subscale. |
| (95) | Cross sectional study | 17 cannabis users and 17 nonusers | Users: 22.4 (1.9)  Nonusers: 23.9 (4.2) | 7.5 % (2.9) THC | Cannabis administration was associated with a mean increase of 8.6 (5.6) points on the Psychotomimetic States Inventory psychotic symptom subscale. |
| (96) | Randomized controlled trial | 11 cannabis users | 21.7 (NR) | 0.9%, 1.4%, and 2.7% THC | Cannabis administration was associated with small but significant increases in acute symptoms of anxiety and confusion. |
| (97) | Naturalistic study | 410 cannabis users | 20.56 (1.68) | 9.60 % (4.69) THC | Use of higher potency cannabis was associated with an increased risk of cannabis dependence, both as measured by self-report and clinician ratings.  Use of high potency cannabis was not associated with an increased risk of psychotic-like symptoms, as measured by either self-report or clinician rating. |
| (98) | Randomized controlled trial | 5 cannabis users | 22 (3.8) | 3.55% THC | Cannabis administration was associated with worsened overall cognitive performance and verbal memory.  Cannabis administration was not associated with any changes to RT, time perception, or number recognition. |
| (99) | Randomized controlled trial | 114 cannabis users | 21.5 (3.3) | 0% or 2.8% THC | Administration of the 2.8% THC cannabis to participants who expected THC was associated with a decrease in anxiety. |
| (100) | Non-randomized experimental study | 44 cannabis users (22 low CBD group, 22 high CBD group) | Low CBD: 21.38 (2.01)  High CBD: 21.55 (1.82) | Low CBD: 6.92% (2.83) THC, 0.08% (0.05) CBD  High cannabidiol: 8.39% (4.76) THC, 4.61% (1.95) CBD | Use of low CBD cannabis was associated with worse delayed and immediate recall as well as increased ratings of anxiety compared to participants using high CBD cannabis.  Use of either cannabis type was associated with a significant acute increase in psychotic symptoms. |
| (101) | Non-randomized experimental study | 94 cannabis users (30 low CBD:THC ratio group, 31 high CBD:THC ratio group) | 21.3 (1.42) | Low CBD:THC ratio group: 11.92% (5.41) THC, 0.14% (5.41) CBD  High CBD:THC ratio group: 7.74% (4.20) THC, 2.64% (2.54) CBD | Participants who used low CBD:THC ratio cannabis displayed an attentional bias toward food- and cannabis-related images whereas those who used high CBD:THC ratio cannabis did not.  Participants who used low CBD:THC ratio cannabis also rated cannabis-related images as being more desirable than those using the high CBD:THC ratio cannabis. |
| (102) | Cross sectional study | 422 cannabis users | NR | Dependent users: 9.56% (4.53) THC, 0.77% (1.65) CBD  Non-dependent users: 9.69% (4.75) THC, 0.88% (1.63) CBD | Cannabis potency was not associated an individual’s number of acute psychotic symptoms at the time of use. |
| (103) | Randomized controlled trial | 122 cannabis and cocaine users** | 22.8 (3.7) | 11-12% THC | Cannabis administration was associated with an increase in impulsive responding and a decrease in processing speed. |
| (104) | Randomized controlled trial | 122 Cannabis, cocaine, and placebo | 22.8 (3.7) | 11-12% THC | Cannabis administration was associated with worsened performance on tasks requiring impulse control, executive functioning, divided attention, and motor control compared to placebo administration. |
| (105) | Randomized controlled trial | 15 cannabis users | 24.3 (s.e. = 1.3) | 5 mg THC | THC administration was associated with:   - an increased likelihood of making risky decisions associated with a neutral outcome, - a decreased likelihood of making risky decisions associated with unsure outcomes, - reduced RT when making risky decisions associated with large payoffs, - and a decreased allocation of attention toward losses on highly risky trials. |
| (106) | Randomized controlled trial | 12 cannabis users | NR | 5.5% THC | Cannabis administration to participants at clinical high risk for psychosis was associated with an increase in psychotic symptoms and deficits in memory and executive functioning. |
| (107) | Randomized controlled trial | 11 cannabis users | 21.7 (2.3) | 6 mg THC | Cannabis administration was not associated with any significant changes in reward processing. |
| (108) | Randomized controlled trial | 21 cannabis and cocaine users | 23 (3.57) | 11% THC | Cannabis administration was associated with a significant increase in all measured symptoms of dissociation compared to placebo administration. |
| (109) | Randomized controlled trial | 28 cannabis users | 22.54 (0.48) | 12.5% (2.0) THC | Cannabis administration was associated with acute increases in symptoms of anxiety and confusion compared to placebo.    Cannabis administration was not associated with any changes in verbal memory, motor control, or attention. |

THC, tetrahydrocannabinol; CBD, cannabidiol; NR, not reported; RT, reaction time; PLE, psychotic-like experience; TTRT, told THC/received THC; TTRP, told THC/received placebo; TPRT, told placebo/received THC; TPRP, told placebo/received placebo.
